# Erectile function after partial penectomy for penile cancer

**DOI:** 10.1590/S1677-5538.IBJU.2019.0119

**Published:** 2020-12-20

**Authors:** Leonardo L. Monteiro, Rodrigo Skowronski, Fadi Brimo, Paulo da C. Carvalho, Romulo A. L. de Vasconcelos, Charley R. C. V. Pacheco, Adriano A. Calado, Wassim Kassouf

**Affiliations:** 1 McGill University Division of Urology Montreal Canada Division of Urology, McGill University, Montreal, Canada.; 2 McGill University Department of Pathology Montreal Canada Department of Pathology, McGill University, Montreal, Canada.; 3 Hospital do Câncer de Pernambuco Departamento de Urologia RecifePE Brasil Departamento de Urologia, Hospital do Câncer de Pernambuco, Recife, PE, Brasil.; 4 Universidade Estadual de Pernambuco Divisão de Urologia RecifePE Brasil Divisão de Urologia, Universidade Estadual de Pernambuco, Recife, PE, Brasil.

**Keywords:** Penile Neoplasm, Erectile Dysfunction, Amputation

## Abstract

**Purpose:**

To evaluate the erectile function in patients who underwent partial penectomy and identify factors associated with penile functional status.

**Materials and Methods:**

We identified patients who underwent partial penectomy due to penile cancer between 2009 and 2014. Clinical and pathological characteristics included patient age at the time of diagnosis, obesity, hypertension, dyslipidemia, diabetes, smoking, metabolic syndrome, Eastern Cooperative Oncology Group (ECOG) status, penile shaft length, tumor size, primary tumor stage (pT), clinical nodal status, and local recurrence. Erectile function was assessed prospectively with the International Index of Erectile Function (IIEF-5) at least 3 months after partial penectomy.

**Results:**

A total of 81 patients met analysis criteria. At the diagnosis, the median age was 62 years (range from 30 to 88). Median follow-up was 17 months (IQR 7-36). Of total patients, 37 (45%) had T2 or higher disease. Clinically positive nodes were present in 16 (20%) patients and seven (8.6%) developed local recurrence. Fifty patients (62%) had erectile dysfunction (ED) after partial penectomy, 30% had moderate or severe erectile dysfunction scores. Patients with ED versus without ED were similar in baseline characteristics except for age, penile shaft length, and presence of inguinal adenopathy (p <0.05). Multivariate analysis using logistic regression confirmed that older patients, shorter penile shaft length, and clinically positive lymph node were significantly associated with ED.

**Conclusion:**

Partial penectomy due to penile cancer provides adequate local control of the disease, however, proper counselling is important especially in relation to ED consequences. Preservation of penile length yields to more optimal erectile recovery.

## INTRODUCTION

Penile cancer is a rare malignancy in North America and Europe with a reported incidence of less than 1 per 100.000 men (1). However, the incidence is higher in some areas of Asia, Africa, and South America. The highest incidence rate of penile cancer is reported in Brazil ranging from 2.9 to 6.8 per 100.000. Nonetheless, there is variability even within Brazil, where the highest incidence is reported in populations living in the Northeast region, reaching 5.7% of the male neoplasias (2,3). Penile cancer has a multifactorial aetiology, and several risk factors have been identified, such as phimosis, smoking, human papilloma virus infection, and chronic inflammatory states (4–6). The vast majority of tumours are squamous cell carcinoma (SCC) (7). The treatment option for penile cancer is complete excision of the tumor with negative margins. Partial amputation is the traditional procedure and is indicated for invasive distal lesions (stages T2-T4) and large T1 tumors (8). The disease itself or the treatment affects corporal image, self-esteem, genital sensibility, and frequently impairing sexual function or micturition. There is a paucity of studies regarding the erectile function of patients undergoing partial penectomy. We hypothesized that partial penectomy due to penile cancer may impair erectile function and clinicopathological characteristics could be correlated with erectile dysfunction (ED). The purpose of this study was to evaluate the erectile function in patients who underwent partial penectomy, and the possible associations between ED and clinicopathological characteristics.

## MATERIAL AND METHODS

Under IRB approval (58147916.1.0000.5205), we reviewed the records of 81 patients who underwent partial penectomy due to penile cancer at two centers, Pernambuco Cancer Hospital and McGill University Health Centre, between 2009 and 2014 that fit the inclusion criteria. Partial penectomy was performed to treat patients with invasive malignance of the penis when organ-sparing surgery was not possible. A guillotine amputation of the penis at least few millimetres proximal to the gross lesion was performed but leaving 1cm of uninvolved urethra protruding from the amputated stump. The inclusion criteria were as follow: histopathological confirmation of penile cancer, Eastern Cooperative Oncology Group (ECOG) Performance Status ≤2, satisfactory baseline sexual intercourse prior to surgery (obtained during interview), and absence of unresectable lymph node and/or distant metastasis. Clinical and pathological characteristic that were collected included patient age at the time of diagnosis, obesity, hypertension, dyslipidemia, diabetes, smoking, metabolic syndrome, ECOG, penile shaft length, tumor size and primary penile tumor stage (pT), clinical status of lymph node, and local recurrence. Staging was performed according to the 7th edition of tumor-node-metastasis (TNM) classification (9). Metabolic syndrome was diagnosed when any three of the following factors were present: triglyceride level ≥150 milligrams per deciliter of blood (mg/dL), HDL cholesterol of ≤40mg/dL, fasting glucose of ≥100mg/dL, blood pressure ≥130/85 millimeters of mercury, body mass index (BMI) ≥30. Erectile function was assessed according to the International Index of Erectile Function (IIEF-5) after partial penectomy. The length of the penis shaft was performed with the penis in a flaccid state, measured in centimeters using a rigid ruler from the pubic bone to the end of the shaft. The possible scores for the IIEF-5 range from 5 to 25, a score of 1- 5 is awarded to each of the 5 questions, and ED was classified into five categories based on the scores: severe (5–7), moderate (8–11), mild to moderate (12–16), mild (17–21), and no ED (22-25). The patients answered the questionnaire only once, at least 3 months after surgery (range from 3 to 6 months).

Continuous variables are presented as median and categorical variables are reported with frequency count and percent. Univariable analysis was performed using the Pearson Chi-Square and Mann-Whitney tests for categorical and numerical outcomes, respectively. The Kaplan-Meier method was applied to estimate the overall and recurrence-free survival. For including the shaft size into the main statistical analyses performed, an outlier test was done, based on the mean and variance of the shaft measurements. Clinicopathological factors were evaluated using univariate and multivariate regression models to find predictors of erectile dysfunction. Variables with a p value <0.6 were entered in the multivariate model (logistic regression), where results with p <0.05 were considered to have statistical significance. The power of the procedure lies in the fact that the variables are introduced into the regression in the order of their significance. A Receiver Operating Characteristics (ROC) curve was also performed to understand the fit of the regression model. The data analysis for this study was generated using the Real Statistics Resource Pack software (Release 5.4).

## RESULTS

**Figure 1:**
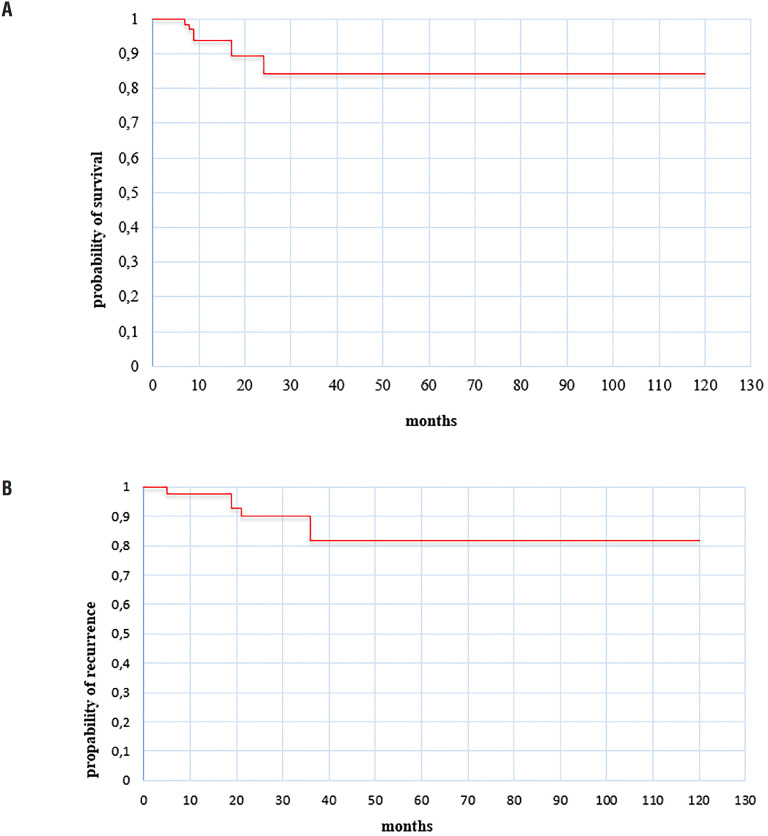
A Kaplan-Maier overall (A) and recurrence-free (B) survival curve.

At the diagnosis, the median age was 62 years (range from 30 to 88). Overall, 37 (45%) had T2 or higher disease and 16 (20%) have clinically positive nodes. Nine patients (11.1%) had a positive surgical margin. After a median follow-up was 17 months (IQR 7-36), seven (8.6%) developed local recurrence. Regarding the clinical characteristics, 18 (22%) of the patients presented metabolic syndrome and 37 (45.7%) were smokers (Table-1). The mean tumor size was 3.35cm±1.53 SD. Fifty patients (61.7%) had erectile dysfunction after partial penectomy. In this group of patients, the degree of ED was mild in 9 (11.2%), mild to moderate in 17 (21%), moderate in 9 (11.2%), and severe in 15 (18.3%). Patients with ED versus those without ED were similar in baseline characteristics except for age, penile shaft length and clinical nodal status (p <0.05, see Table-2). Five-year overall and recurrence-free survival rates were 84% and 82%, respectively (Figure-1). On multivariate analysis, older age (OR 1.12, 95% CI 1.04-1.19, p=0.001), higher postoperative penile shaft length (OR 0.39, 95% CI 0.17-0.87, p=0.021), and clinically positive lymph node (OR 3.34, 95% CI 1.12-9.94, p=0.020) were significantly associated with erectile dysfunction. The ROC curve demonstrates that the fit of the model was very accurate with an Area Under the Curve (AUC) of 0.88 for ED.

**Table 1:** Baseline clinical and pathological characteristics.

No. pts		81
Median age at diagnosis		62 (30 – 88)
Median ± SD residual penile shaft length (cm)		5 ± 1.57 (3 – 10.5)
Mean ± SD tumor size (cm)		3.3 ± 1.53
**Pathological T stage, Nº pts (%):**		
	Ta	2 (2.5)
	T1	42 (51.8)
	T2	35 (43.2)
	T3	2 (2.5)
**Clinically positive lymph nodes, Nº pts (%):**		
	Yes	23 (28.4)
	No	58 (71.6)
**Positive surgical margins, Nº pts (%)**		
	Yes	9 (11.1)
	No	72 (88.9)
**Local recurrence, Nº pts (%):**		
	Yes	7 (8.6)
	No	74 (91.4)
**BMI ≥ 30, Nº pts (%):**		
	Yes	24 (29.6)
	No	57 (70.4)
**Hypertension, Nº pts (%):**		
	Yes	35 (43.2)
	No	46 (56.8)
**Dyslipidemia, Nº pts (%):**		
	Yes	31 (38.3)
	No	50 (61.7)
**Diabetes, Nº pts (%):**		
	Yes	11 (13.6)
	No	70 (86.4)
**Metabolic Syndrome, Nº pts (%):**		
	Yes	18 (22.2)
	No	63 (77.8)
**Smoker, Nº pts (%):**		
	Yes	37 (45.7)
	No	44 (54.3)

**SD** = Standard deviation; **T** = Tumor; **Nº pts** = Number of patients; **BMI** = Body mass index.

**Table 2:** Clinical and pathological differences between patients without and with ED.

Variables		No. Pts without ED (%)	No. Pts with ED (%)	p Value
Nº. pts		31 (38.3)	50 (61.7)	
Age years (median)		54	64	0.0037
Penile Shaft Length, cm (median)		5.5±1.1	4.5±1.8	0.0004
Tumor size, cm (mean ± SD)		3.49±0.85	3.26±1.84	0.07
**Metabolic Syndrome**				0.06
	Yes	06 (9.4)	24 (24.0)	
	No	25 (80.6)	38 (76.0)	
**Obesity (BMI ≥30)**				0.056
	Yes	13 (41.9)	11 (22.0)	
	No	18 (58.1)	39 (78.0)	
**Hypertension**				0.229
	Yes	16 (51.6)	19 (38.0)	
	No	15 (48.4)	31 (62.0)	
**Dyslipidemia**				0.315
	Yes	14 (45.2)	17/50 (34.0)	
	No	17 (54.8)	33/50 (66.0)	
**Diabetes**				0.089
	Yes	03 (09.7)	08 (16.0)	
	No	28 (90.3)	42 (84.0)	
**Smoker**				0.941
	Yes	14 (45.2)	23 (46.0)	
	No	17 (54.8)	27 (54.0)	
**Local recurrence**				0.58
	Yes	02 (6.5)	05 (10.0)	
	No	29 (93.5)	45 (90.0)	
**Pathological T stage**				0.321
	< T2	19 (61.3)	25 (50)	
	≥ T2	12 (38.7)	25 (50)	
**Positive surgical margins**				
	Yes	4 (12.9)	5 (10.0)	0.686
	No	27 (87.1)	45 (90.0)	
**Clinically positive lymph nodes**				0.015
	Yes	4 (12.9)	19 (38)	
	No	27 (87.1)	31 (62)	

1 **ED** = Erectile dysfunction; **Nº pts** = Number of patients; **SD** = Standard deviation; **BMI** = Body mass index; **T** = Tumor.

## DISCUSSION

Penile cancer is most common in developing countries where it accounts for up to 10% of cancers in men (1). It has significant physiological as well as psychological effects on the patient. These effects arise from the cancer diagnosis itself and the treatment consequences. Our study demonstrated that partial penectomy provides excellent local control of the disease with acceptable maintenance of sexual function.

Partial penectomy provides local control of the disease while decreasing extent of disfiguration (10, 11). Horenblas published in 2014 the experience of 56 years in surgical treatment of penile cancer from a single center (12). In this retrospective study, 408 patients underwent a partial amputation, 29% of them experienced recurrent disease during follow-up of 65 months, including only local recurrence in 4%, locoregional in 4%, local and distance recurrence in 1%, and regional and/or distant recurrence in 20%. The overall survival rate was 67% (95% CI 64-70). Five-year CSS was 81% (95% CI 78-84). For early stage penile cancer, pathological T1/T2, a National Cancer Database analysis reported the largest cohort on the survival outcomes of the different surgical approaches. The majority of the surgical procedures offered to patients were partial penectomy, corresponding to 2.360 (56.79%) patients. In this group, most patients had a tumor size <3cm (59.7%), and the positive margin rate was 7.2%. The 3-, 5- and 10 year overall survival rates were 92%, 87% and 73%, respectively (13). In our cohort, similar observations were noted regarding 5-year overall and recurrence-free survival 84% and 82%, respectively, mean tumor size of 3.35±1.53cm, and local recurrence in 8.6% of patients. These outcomes reinforce the therapeutic role of partial penectomy in patients with localized invasive penile cancer.

Maintenance of adequate sexual function is a very common concern for men undergoing treatment for penile cancer. Patients treated for penile cancer by various methods reported that they would choose a treatment with lower long-term survival if it increases the chances of remaining sexually potent (14). Few studies had evaluated the effects of partial penectomy on erectile function for penile cancer patients. While partial penectomy may reduce the length of the penis by more than 25%, there may still be sufficient residual tissue for successful vaginal penetration (15). Romero (16) assessed 18 patients pre- and post-operatively using IIEF questionnaire in patients who underwent partial penectomy due to penile cancer. They observed that only 55.6% of patients reported erectile function of the penile stump that allowed sexual intercourse. The rest of the patients had significantly reduced erectile function after surgery. One of the largest cohort (n=36) of patients who underwent partial penectomy showed IIEF scores were 50% lower on erectile function when compared with healthy men with a mean IIEF score for erectile function of 11.30±9.32 (17). Recently, a prospective study with 43 patients reported statistically significant reduction of erectile function after partial penectomy where IIEF-15 score decreased from 26.70±3.07 to 17.81±10.66 (18). Age was negatively associated with erectile function (19). A multicenter study by Sansalone (20) enrolled 25 patients who underwent partial penectomy due to penile cancer. They reported significant decrease of erectile function postoperatively; IIEF mean score decreased from 28.68±1.04 to 21.28±3.07 (p <0.001). Another small study of 10 patients treated with partial penectomy and reconstruction with ventral fenestrated flap technique demonstrated the average IIEF score in the preoperative period was 21.6, one month postoperatively was 13, and 40 months postoperatively was 19.7 (mild erectile dysfunction) (21). In 2004, Rempelakos reported the largest cohort in which sexual function after partial penectomy was assessed (22). From 227 patients, only 26% of patients maintained satisfactory erection. In contrast, two small studies showed excellent different outcomes regarding erectile function after partial penectomy (11, 23). Perhaps the preoperative evaluation of patients at a date very close to surgery represents the moment of greater anxiety and activity of the disease and, consequently, explains this result. Age was a significant predictor for worse sexual potency after surgery (24).

Our analyses showed that most patients, approximately 62% of the patients had erectile dysfunction after partial penectomy due to penile cancer. However, only 30% of patients presented moderate or severe erectile dysfunction after the procedure. Approximately 50% of them retained satisfactory erectile function, patients with IIEF score ≥21 (mild or no erectile dysfunction). Risk factors for erectile dysfunction such as obesity, hypertension, dyslipidemia and smoking between patients with and without erectile dysfunction was not significantly different. The logistic regression performed confirmed the importance of age, shaft size and the presence of clinically positive lymph nodes in association with erectile dysfunction. Older patients were negatively associated with erectile function that is consistent with previous study (20). Penile shaft length was associated with erectile function after partial penectomy; smaller shaft length increased possibility of erectile dysfunction. The exact cause of erectile dysfunction among men with clinically positive nodes is uncertain but may be multifactorial including anxiety or depression related to advanced stage, the burden of additional therapy or other yet unexplained factors that require further study.

To our knowledge, this is the second largest study regarding erectile function in patients who underwent partial penectomy due to penile cancer. Besides, this is the first study that combines clinical and pathological characteristics in identifying factors associated with erectile dysfunction in the patients. Our study confirms that partial penectomy due to penile cancer provides good oncological control of the disease. Moreover, patients can retain erectile function if they have early diagnosis and lower volume of disease especially in younger patients with localized disease and longer residual penile shaft length after surgery. The scarce literature available on the effects of partial penectomy on erectile function for penile cancer patients is listed in Table-3.

**Table 3:** Erectile function after partial penectomy.

Author	Questionnaire	Number of patients	Pre-op (mean)	Pos-op (mean)	p-value
Rempelakos et al., 2004 (22)	No data	227	No data	26% satisfactory	
Yu et al., 2016 (18)	IIEF	43	26.70±3.07	17.81±10.66	< 0.01
Kieffer et al., 2014 (17)	IIEF	36	No data	11.30±9.32	
Sansolone et al., 2017 (20)	IIEF	25	28.68±1.04	21.28±3.07	< 0.001
Romero et al., 2005 (16)	IIEF	18	* 29.56±1.42	* 19.39±12.44	< 0.012
Santos-Lopes et al., 2018 (19)	IIEF	16	23.4±4,4	16.6±0.001	< 0.001
D’Ancona et al., 1997 (23)	OSFQ	14	100% normal	64% normal / slightly decreased	
Alei et al., 2012 (21)	IIEF	10	21.6	19.7	
Wan et al., 2018 (11)	IIEF	8	11.75±1.83	20.38±2.26	< 0.05
Present study, 2018	IIEF	81	100% satisfactory erections	16.18±7.08	

* Median; **IIEF** = International index of erectile function; **OSFQ** = Overall sexual functioning questionnaire.

Limitations to our study include retrospective approach with its inherent selection bias and unmeasured confounding variables. Further, we did not measure psychological problems, such as depression and anxiety, which are risk factors for erectile dysfunction.

## CONCLUSION

Partial penectomy for penile cancer provides adequate control of the disease, however, proper counselling is important especially in relation to ED consequences. Preservation of penile length yields to more optimal erectile recovery.
